# Genome-Wide Identification of the *BXL* Gene Family in Soybean and Expression Analysis Under Salt Stress

**DOI:** 10.3390/ijms26199534

**Published:** 2025-09-29

**Authors:** Yimin Wen, Biwei Lai, Weijie Hu, Mengyang You, Lingshuang Wang, Tong Su

**Affiliations:** 1 Guangdong Provincial Key Laboratory of Plant Adaptation and Molecular Design, Innovative Center of Molecular Genetics and Evolution, School of Life Sciences, Guangzhou University, Guangzhou 510006, China; 13711483099@163.com (Y.W.); lbw84054593@163.com (B.L.); 13556172449@163.com (W.H.); 2 School of Environmental Science and Engineering, Guangzhou University, Guangzhou 510006, China; myyou@gzhu.edu.cn

**Keywords:** gene family, β-D-xylosidase, expression pattern, salt, soybean

## Abstract

β-D-xylosidases (BXLs) are pivotal enzymes in xylan degradation, playing essential roles in plant development and stress responses. In this study, we identified 29 *GmBXL* genes in soybean through homolog alignment. Phylogenetic analysis classified these genes into three groups, with Group III being legume-specific. The *GmBXLs* are unevenly distributed across 15 chromosomes, with their expansion driven by both tandem and segmental duplications. Conserved motif and domain analyses revealed functional conservation, particularly in family 3 of glycoside hydrolase domains. Promoter regions of *GmBXLs* are enriched with hormone-responsive and stress-related *cis*-elements, indicating their involvement in diverse biological processes. Tissue-specific expression analysis revealed differential *GmBXLs* expression across leaves, roots, flowers, and seeds, with *GmBXL13* and *GmBXL26* exhibiting notably high transcript levels in pods and seeds. Under salt stress, 26 *GmBXLs* exhibited significant expression changes, with 20 genes up-regulated in both leaves and roots, highlighting their roles in salt tolerance. These findings enhance our understanding of the evolutionary and functional characteristics of *GmBXLs*, providing valuable insights for molecular breeding of salt-tolerant soybean varieties.

## 1. Introduction

The plant cell wall serves as a crucial interface for environmental signal perception and transduction, facilitating adaptive responses to external stimuli. This dynamic structure is primarily composed of three major polysaccharide components: cellulose, hemicellulose, and pectin [1,2]. Among these components, hemicellulose predominantly comprising xylan and xyloglucan polymers, represents the second most abundant carbohydrate fraction in the cell wall [3]. During the maturation of the cell wall, xylan undergoes hydrolysis under the action of a series of enzymes to form xylose. β-D-xylosidase (BXL; EC3.2.1.37) plays a pivotal role in this process as the key enzyme responsible for the terminal degradation of xylan [4]. Its activity is essential for completing the xylan catabolic pathway and facilitating subsequent metabolic utilization of the released xylose units.

Plant BXLs are predominantly classified into glycoside hydrolase family 3 (GH3) based on their substrate specificity, amino acid sequence conservation, three-dimensional structural features, and catalytic mechanisms [5,6]. These enzymes typically contain three characteristic domains: an N-terminal catalytic domain (PF00933), a C-terminal catalytic domain (PF01915), and a fibronectin type III domain (PF14310) whose biological function remains to be elucidated [7,8]. The N-terminal domain harbors two conserved substrate-binding motifs: a Tryptophan-Glycine-Arginine (WGR) motif and a Lysine-Histidine (KH) motif [3]. Mechanistically, BXLs employ an acid-base catalytic mechanism for xylan hydrolysis, with two essential catalytic residues at the active site: (i) a nucleophilic aspartate (Asp) residue and (ii) an acid-base glutamate (Glu) residue [9].

Currently, BXLs have been identified and functionally characterized in multiple plant species. In *Arabidopsis thaliana*, seven *AtBXL* genes have been identified, with *AtBXL1* showing high stem-specific expression whose mutation results in shortened stems, reduced silique length and decreased seed production [10], while its expression is upregulated under darkness or sugar starvation conditions [11,12]. AtBXL4 accumulates in the apoplast during systemic acquired resistance (SAR) and serves as a key component in the SAR signaling pathway [13]. In fruit ripening, *Pyrus pyrifolia JPRXYL* is associated with senescence and accumulates in ripened fruits and old leaves [14], while peach *PpXy1* functions as a molecular indicator for woolliness tolerance and helps fruits cope with chilling stress [15]. *FaXyl1* exhibits down-regulated expression upon gibberellin A3 and 1-naphthaleneacetic acid treatment, with its promoter harboring multiple hormone-responsive elements in *Fragaria ananassa* [16]. *Brassica juncea* possesses 16 *BjuBXL* genes, among which *BjuBXL1-1* and *BjuBXL1-2* are crucial for stem swelling [17]. In *Populus trichocarpa*, the expressions of *PtBXL4*, *PtBXL5*, *PtBXL9* are induced under low and high ammonium and nitrate treatment [18]. Importantly, crop BXLs participate in abiotic stress responses, such as *OsBXL1* and *OsBXL3* respond to salt-alkali stress in rice [19]. Overall, these studies underscore the multifaceted roles of *BXL* genes in plant development, stress adaptation, and hormone responses across diverse species. However, systematic identification and comprehensive functional analysis of the *BXL* gene family in soybean remain largely unexplored.

Soybean (*Glycine max* L.), a globally important legume crop, serves as a vital source of plant-based proteins and edible oils for human nutrition. However, increasing soil salinization has emerged as a major constraint significantly reducing soybean productivity worldwide. Salt stress induces multiple physiological damages in plants, including cellular dehydration, nutrient imbalance, and excessive accumulation of reactive oxygen species (ROS) that cause oxidative stress and tissue damage [20,21]. Soybean has evolved diverse regulatory mechanisms to adapt to salt stress. Ion transporters help remove excessive NaCl from the cell, thereby alleviating the effects of salt stress [22]. The cation/proton antiporter (CPA) gene, *GmCHX1*, which is highly expressed in the root vasculature, helps restrict Na^+^ loading into the shoot [23,24,25]. Another CPA gene, *GmCHX20a* contributes to enhanced salt tolerance during subsequent salt stresses [26]. *GmSOS1* (Salt Overly Sensitive 1), which encodes a Na^+^/H^+^ exchanger, is salt-responsive and shows dosage-dependent expression in roots [27]. Members of the high-affinity K^+^ transporter (HKT) family reduce Na^+^ accumulation and improve K^+^ uptake under salt treatment [28,29]. The highly hydrophilic of the late embryogenesis abundant (LEA) proteins is an important characteristic for osmoregulation; for instance, *GmLEA2-1* improves salt tolerance in soybean [30]. In addition, multiple genes underpinning the production of antioxidants and ROS scavenging pathways have been identified. Purple acid phosphatase 3 (GmPAP3) reduces ROS levels and enhances ascorbic acid-like antioxidation pathway activities [31,32]. Salt stress promotes the activation of numerous transcription factors, such as bHLH, bZIP, and AP2/ERF families, and initiates multiple signal transduction pathways, including those mediated by phytohormones and phosphatidylinositol signaling [33,34,35]. Furthermore, plants modulate the abundance, composition, and distribution of cell wall components to sustain growth under prolonged stress [36,37]. For example, a co-chaperone DNAJ protein, *GmDNAJC7* upregulates genes related to cellulose biosynthesis [38].

Considering the crucial roles of *BXL* genes in plant cell walls and their demonstrated involvement in abiotic stress responses across species, we speculate that GmBXLs may participate in soybean salt stress response. Therefore, in this study, we performed genome-wide identification and characterization of the *BXL* gene family in soybean. Through systematic homology analysis, we identified 29 *GmBXL* genes and conducted comprehensive investigations of their physicochemical properties, phylogenetic relationships, conserved protein motifs, gene structures, and expression profiles. This study provides fundamental genomic resources for future functional studies of salt-tolerance genes and facilitates molecular breeding efforts to improve soybean adaptation to saline environments.

## 2. Results

### 2.1. Identification of the Soybean BXL Genes

To systematically identify members of the *BXL* gene family in soybean, we performed a comprehensive genome-wide analysis using seven well-characterized *A. thaliana* BXL protein sequences as queries for BLASTP searches. Candidate genes were further verified using both the Pfam database and Conserved Domain Database (CDD) to ensure accurate identification of functional domains. This rigorous screening process identified 29 high-confidence *GmBXL* genes in the soybean genome (Table S1). Detailed characterization of these GmBXL proteins revealed considerable variation in their physicochemical properties (Table S3). The proteins ranged in size from 502 to 802 amino acids, corresponding to molecular weights between 54.80 and 86.32 kDa. Theoretical isoelectric points (pI) varied from 5.26 to 9.26, suggesting potential functional diversity in different cellular environments. All proteins demonstrated good stability, with instability indices between 22.75 and 38.35, while aliphatic indices ranged from 84.63 to 95.68, indicating thermostability. Hydrophobicity analysis showed that most GmBXL proteins (24 out of 29) are hydrophilic (GRAVY < 0), with only GmBXL7, 15, 19, 20, and 26 exhibiting hydrophobic characteristics. Subcellular localization predictions revealed GmBXLs are distributed in different organelles: eighteen proteins were predicted to localize in mitochondria, six in chloroplasts, four in the cytoplasm, and one in peroxisomes. These diverse physicochemical characteristics and subcellular distributions strongly suggest that *GmBXL* family members have evolved specialized functional roles in various cellular processes.

**Figure 1:**
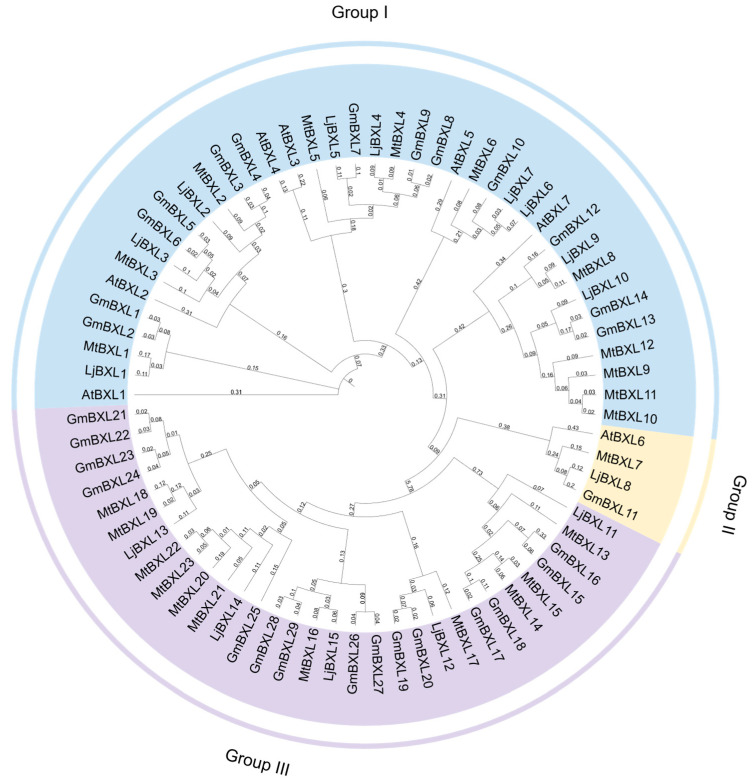
Phylogenetic tree of *Arabidopsis* and legumes BXLs. The sequences of published BXLs in *Arabidopsis thaliana*, *Glycine max*, *Medicago truncatula*, and *Lotus japonicus* were obtained from Phytozome 13 (https://phytozome-next.jgi.doe.gov/, accessed on 15 July 2025) [39]. The corresponding amino-acid sequences were aligned using CLUSTALW to create multiple alignments on MEGA11 [40]. The phylogenetic tree was constructed using IQ-TREE [41] under the best-fit substitution model Q.plant + R5 [42]. Log-likelihood of the tree: −39,608.0998 (s.e. 821.0322). Unconstrained log-likelihood (without tree): −5860.0545. Number of free parameters (#branches + #model parameters): 153. Akaike information criterion (AIC) score: 79,522.1996; Corrected Akaike information criterion (AICc) score: 79,588.1996. Bayesian information criterion (BIC) score: 80,251.4269. Total tree length (sum of branch lengths): 21.2530. Sum of internal branch lengths: 14.2670 (67.1291% of tree length). All proteins were categorized into three clades; the accession numbers of these genes are listed in Table S1.

To elucidate the evolutionary relationships among BXL proteins, we performed comprehensive phylogenetic analysis using 74 protein sequences from four plant species: *A. thaliana*, *G*. *max*, *M*. *truncatula*, and *L*. *japonicus*. The neighbor-joining phylogenetic tree constructed from multiple sequence alignments revealed clear clustering of BXL proteins into three distinct groups (Figure 1). Group I emerged as the largest clade, containing the majority of BXL members, while Group II represented the smallest group with only four conserved members. Notably, Group III appears to be a legume-specific clade, comprising 31 members exclusively from the three legume species, including 23 from *M*. *truncatula* and 15 from *L*. *japonicus*. This phylogenetic distribution suggests both functional conservation and diversification during the evolution of *BXL* genes, with the legume-specific Group III potentially representing lineage-specific functional specialization.

### 2.2. Chromosomal Distribution and Colinearity Analysis of Soybean BXL Genes

**Figure 2:**
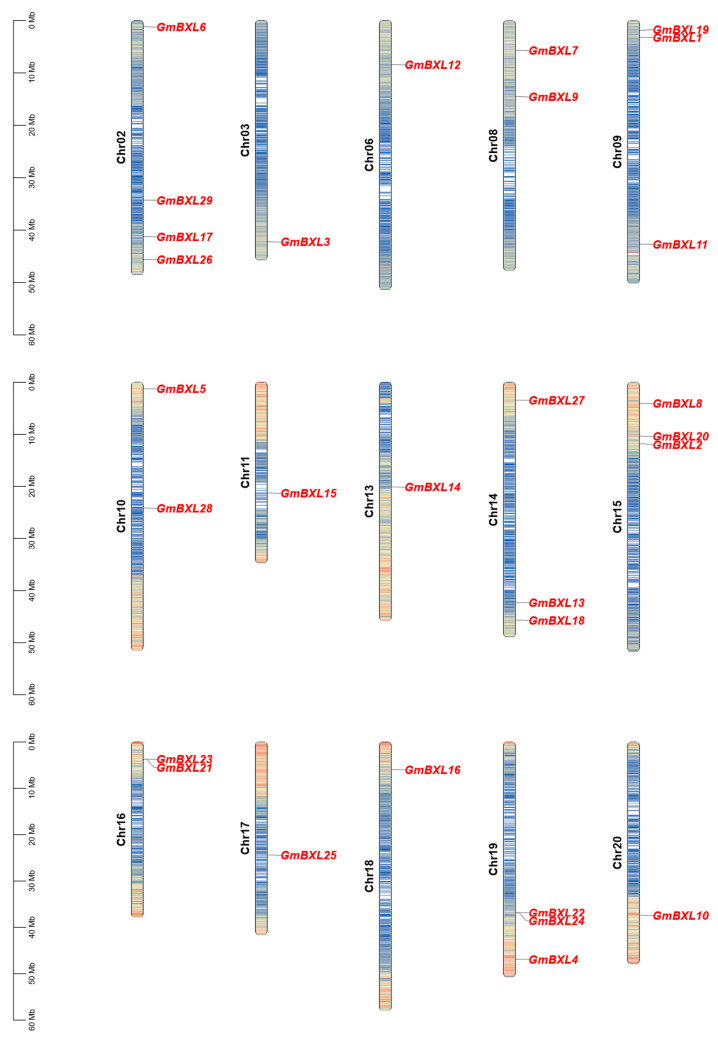
The distribution of soybean *BXL* genes across the chromosomes. The positions of *GmBXL* genes are mapped on their corresponding chromosomes. The vertical scale (left) indicates chromosomal length in millions of base pairs (Mb). The color on the chromosomes indicates the density of genes.

Chromosomal localization analysis revealed that the 29 identified *GmBXL* genes are distributed unevenly across 15 chromosomes (Figure 2), with the majority preferentially located in the end region of the chromosome while *GmBXL14*, *GmBXL15*, *GmBXL25*, and *GmBXL28* were uniquely positioned near centromeric regions. The distribution pattern showed single gene occurrences on chromosomes Chr03, Chr06, Chr11, Chr13, Chr17, Chr18, and Chr20; two genes each on Chr08, Chr10, and Chr16; three genes on Chr09, Chr14, Chr15, and Chr19; and *GmBXL6*, *GmBXL17*, *GmBXL26*, and *GmBXL29* on Chr02, demonstrating a non-random genomic organization that may reflect evolutionary and functional specialization within this gene family.

**Figure 3:**
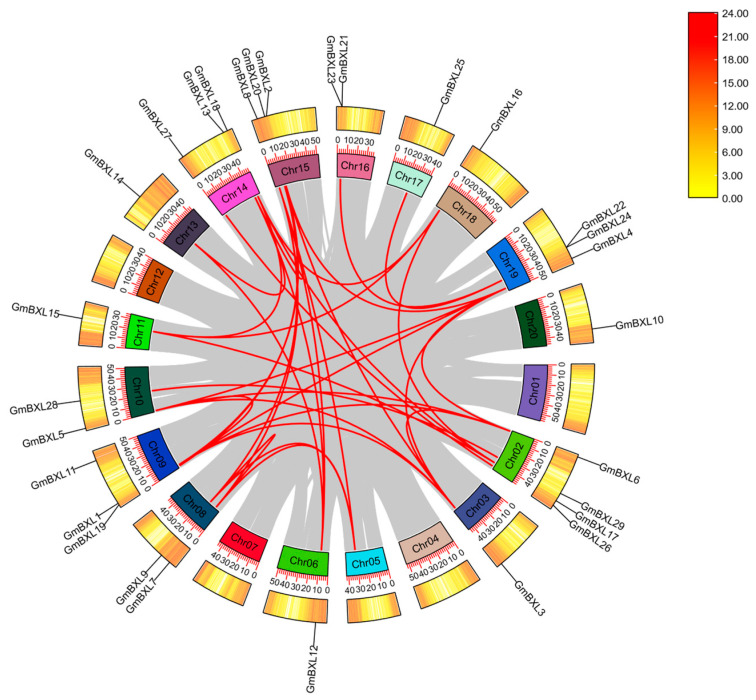
Chromosomal distribution and inter-chromosomal relationships of *GmBXL* genes in soybean. Gray lines represent the gene pairs in the whole genome. The numerical scale along each chromosome indicates the position in megabases (Mb), providing a reference for gene localization. The heatmaps adjacent to each chromosome illustrate the density or intensity of a particular genomic feature. The color gradient in the heatmap, ranging from yellow to red, corresponds to the legend on the top right corner, where yellow represents lower values and red indicates higher values. The red lines highlight collinear relationships (syntenic gene pairs) within the *GmBXL* gene family.

Gene duplication makes it possible to expand gene families, encompassing tandem and segmental duplications, which result from recombination or DNA replication and whole-genome duplications [43,44]. We noticed that *GmBXL21/23* and *GmBXL22/24* are tandem duplications on Chr16 and Chr19, respectively (Figure 2). Furthermore, colinearity analysis showed 36 segmental duplications involving in *GmBXL* genes across the soybean genome (Figure 3). These findings demonstrate that the evolutionary expansion of the *BXL* gene family in soybean has been driven by complementary mechanisms of both tandem and segmental duplication, contributing to the functional diversification of this important enzyme family.

### 2.3. Analysis of Gene Structures, Conserved Motifs, and Domains

**Figure 4:**
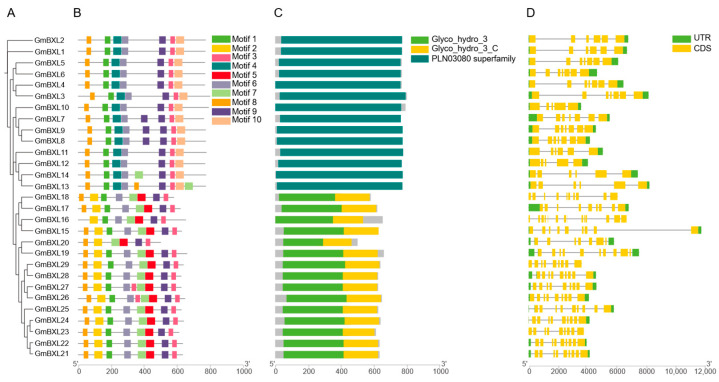
Analysis of conserved motifs, protein domains and gene structures of *GmBXL* genes. (**A**) The phylogenetic tree was constructed using IQ-TREE [41] under the best-fit substitution model WAG + G4 [42]. Log-likelihood of the tree: −19,185.8304 (s.e. 352.6312). Unconstrained log-likelihood (without tree): −5704.1245. Number of free parameters (#branches + #model parameters): 56. Akaike information criterion (AIC) score: 38,483.6608; Corrected Akaike information criterion (AICc) score: 38,491.7011. Bayesian information criterion (BIC) score: 38,749.4599. Total tree length (sum of branch lengths): 9.5957. Sum of internal branch lengths: 7.3326 (76.4156% of tree length). (**B**) The phylogenetic relationship among GmBXL members, along with 10 identified conserved protein motifs. (**C**) Three conserved protein domains of GmBXLs were identified using CDD, with each domain represented in different colors. The scale bars at the bottom indicate protein length. (**D**) The gene structures of *GmBXL* genes with yellow boxes representing coding sequences (CDS), black lines indicating introns, and green boxes representing untranslated regions (UTRs). The scale bar indicates the genomic sequences lengths.

Conserved protein motif analysis contributes to elucidating gene function. In the soybean *BXL* gene family, we identified 10 distinct protein motifs (Figure 4A and Figure S1). Comparative analysis revealed that Group I and Group II members share similar arranged order motifs and both contain the characteristic β-xylosidase superfamily domain (PLN03080) (Figure 4B,C) [45]. In contrast, Group III proteins exhibit a unique configuration comprising eight conserved motifs (1, 2, 3, 5, 6, 7, 8) along with Glyco_hydro_3 and Glyco_hydro_3_C domains. Notably, exon-intron structure analysis demonstrated considerable variation in gene structures across all *GmBXL* members, with no obvious rules found among the Groups (Figure 4C). These structural variations in conserved motifs and gene organization suggest functional diversification within the soybean *BXL* gene family, providing important clues for understanding their potential roles in various biological processes.

### 2.4. Analysis of Cis-Elements in BXL Gene Promoters

**Figure 5:**
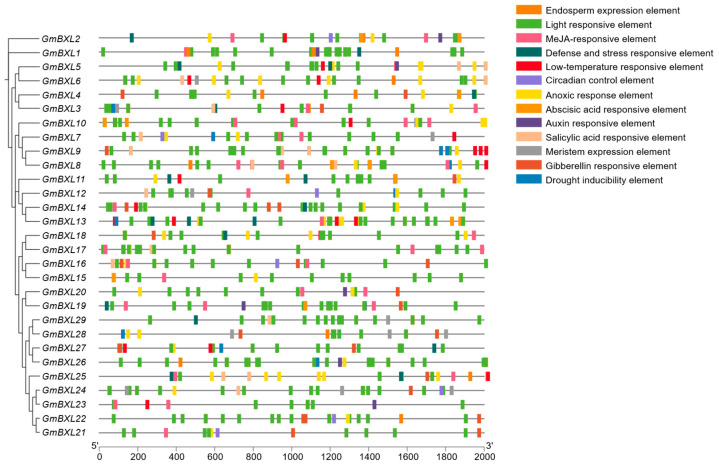
*Cis*-element analysis of *GmBXL* promoters in soybean. Color-coded boxes denote distinct *cis*-element types, as identified on the right. The scale bar at the bottom indicates promoter sequence length (base pairs).

*Cis*-acting elements are important in the transcriptional regulation of gene expression. To investigate potential regulatory mechanisms of *GmBXL* genes, we analyzed 2 kb upstream sequences using PlantCARE, identifying 13 distinct *cis*-regulatory elements associated with abiotic and biotic stress responses (Figure 5). Light-responsive elements were the most abundant, present in all *GmBXL* promoters, with *GmBXL26* containing a remarkable 22 such elements (Table S4). Hormone-responsive elements were also prevalent, including those for MeJA, abscisic acid, gibberellin, salicylic acid, and auxin signaling. Additionally, some anoxic response elements, as well as defense- and stress-responsive elements, and low-temperature responsive elements were found in the promoters of *GmBXLs*. In contrast, elements associated with circadian control, drought inducibility, meristem-specific expression, and endosperm expression were relatively rare, suggesting limited involvement of *GmBXL* genes in these particular biological processes. This comprehensive *cis*-element profiling indicates that *GmBXL* family members likely participate in diverse physiological processes critical for soybean growth and environmental adaptation, particularly those related to light signaling and hormonal regulation.

### 2.5. Response of BXL Genes Under Salt Stress

**Figure 6:**
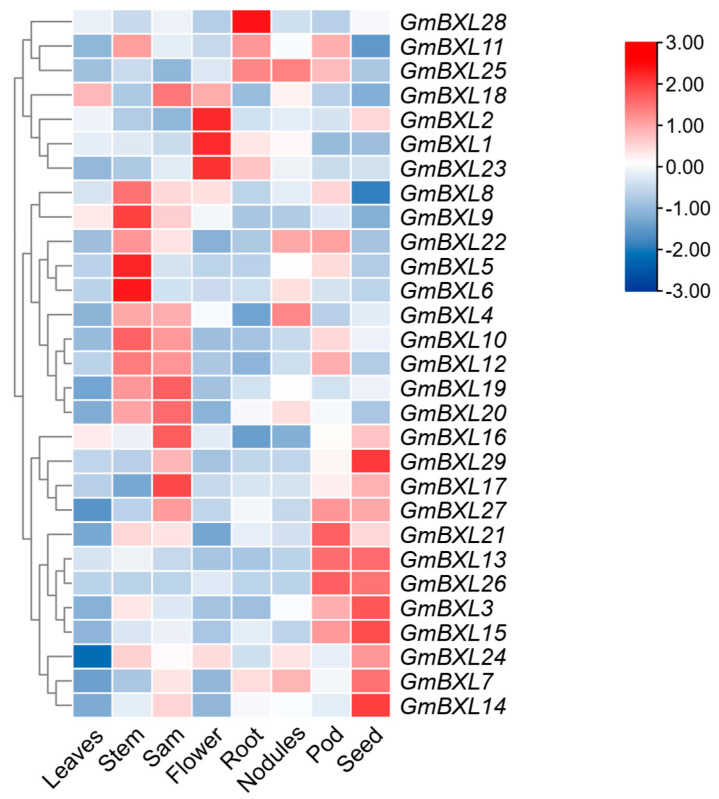
Expression analysis of *GmBXL* genes across soybean tissues. The expression level of *GmBXL* genes were normalized as log2^FPKM+1^ and visualized in a heatmap. A color gradient from blue to red represents expression levels, with blue indicating low expression and red indicting high expression. A dendrogram on the left side of the heatmap illustrates the hierarchical clustering of *GmBXL* genes based on their expression patterns across different tissues.

To investigate the spatial expression profiles of *GmBXL* genes, we analyzed transcriptome data obtained from the Phytozome database originally generated by the Gary Stacey laboratory across eight soybean tissues: leaves, stems, shoot apical meristems (sam), flowers, roots, nodules, pods, and seeds (Figure 6). The analysis revealed distinct tissue-specific expression patterns among *GmBXL* family members. Notably, *GmBXL1*, *GmBXL2*, and *GmBXL23* showed predominant expression in flowers, while *GmBXL5*, *GmBXL6*, and *GmBXL8* exhibited particularly high expression in stems. *GmBXL13* and *GmBXL26* displayed strong preferential expression in pods and seeds, suggesting their potential functional specialization in seed development processes. The majority of *GmBXL* genes demonstrated relatively high expression levels in both roots and leaves, with *GmBXL28* in particular showing extremely high expression levels in roots and leaves. These diverse expression patterns indicate functional diversification within the *GmBXL* gene family, with different members potentially playing specialized roles in various plant organs and developmental processes.

**Figure 7:**
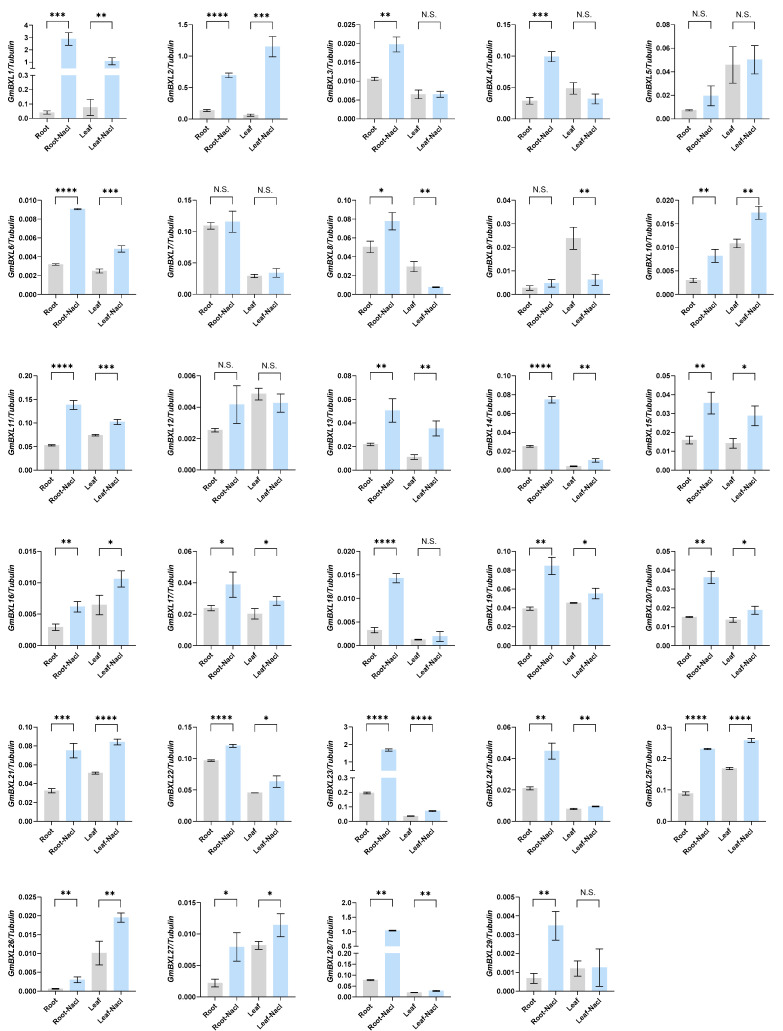
RT-qPCR validation of *GmBXL* genes under salt stress. Plants at the V1 developmental stage were treated with either 200 mM NaCl or a NaCl-free nutrient solution (control). Following four hours of treatment, samples were collected and expression changes in *GmBXL* genes were analyzed using RT-qPCR. Transcript levels of *GmBXL* genes were determined in roots and leaves. The differential expression analysis was conducted based on the 2^−∆∆ct^ method. Relative expression was normalized to *Tubulin* and data are the mean ± SD of three biological replicates. Student’s *t* tests were used to determine statistical significance (* *p* < 0.05; ** *p* < 0.01; *** *p* < 0.001; **** *p* < 0.0001; N.S. indicates no significant difference).

Soil salinity is a major yield-limiting factor in soybean production [46], prompting our investigation of *GmBXL* gene responses to salt stress. We treated plants at the V1 development stage with either 200 mM/L NaCl or a NaCl-free nutrient solution (control). Samples were collected after four hours of treatment and expression changes in *GmBXLs* were analyzed through RT-qPCR. Our results demonstrate that 26 of 29 *GmBXL* genes exhibited significant expression changes under salt stress (Figure 7), with *GmBXL1*, *GmBXL2*, *GmBXL6*, *GmBXL10*, *GmBXL11*, *GmBXL13*, *GmBXL14*, *GmBXL15*, *GmBXL16*, *GmBXL17*, *GmBXL19*, *GmBXL20*, *GmBXL21*, *GmBXL22*, *GmBXL23*, *GmBXL24*, *GmBXL25*, *GmBXL26*, *GmBXL27*, and *GmBXL28* showing coordinated up-regulation in both leaves and roots, suggesting conserved roles in systemic salt tolerance through potentially shared regulatory pathways. Notably, *GmBXL3*, *GmBXL4*, *GmBXL18*, and *GmBXL29* displayed root-specific induction, implicating their specialized function in root-based stress adaptation, while *GmBXL8* and *GmBXL9* showed marked leaf-specific down-regulation, indicating distinct regulatory mechanisms in aerial tissues compared to other family members. These differential expression patterns reveal both coordinated and tissue-specific regulation of *GmBXL* genes in soybean salt stress response, highlighting their functional diversification in abiotic stress adaptation.

## 3. Discussion

Salt stress adaptation is a critical ecological trait enabling plants to survive in saline-alkali environments. BXLs plays a pivotal role in xylan degradation and have been implicated in plant stress responses, particularly salt tolerance. In this study, 29 *GmBXL* genes were identified in the soybean genome and classified into three groups. Motif analysis reveals that members of Group I and Group II exhibit similar motif compositions (Figure 4), whereas Group Ⅲ possesses distinct motifs containing both Glyco_hydro_3 and Glyco_hydro_3_C domains. Furthermore, the promoters of most *GmBXL* genes are enriched with hormone-responsive *cis*-elements (Figure 5). Under salt stress, 26 *GmBXL* genes showed significant expression changes, displaying coordinated or antagonistic regulatory patterns, which supports their functions involvement in soybean salt response.

Gene duplication is a fundamental evolutionary mechanism that generates genetic novelty across all life forms [47,48,49,50]. This process occurs through various mechanisms including tandem and segmental duplications arising from recombination or DNA replication and the whole-genome duplications [44]. Our analysis reveals that the *BXL* gene family in soybean has expanded through both tandem and segmental duplications (Figure 2 and Figure 3). By comparing the functional characteristics of evolutionarily young and old duplicated gene pairs, we gained insights into the patterns of functional conservation and divergence within this gene family.

In evolutionary analysis, members belonging to the same evolutionary branch usually exhibit functional similarity. *AtBXL1* shows stem-specific expression, the mutation of whose results in reduced stem and silique length [10]. In soybean, the evolutionarily closest homologs *GmBXL1* and *GmBXL2* display predominant expression in flowers with relatively high expression in stems (Figure 6), suggesting potential functional conservation in stem development while acquiring specialized roles in floral biology. Functional predictions based on phylogenetic relationships further reveal that *GmBXL7*, *GmBXL8*, and *GmBXL9*—orthologs of *AtBXL4*—likely participate in cell wall polysaccharide metabolism. These enzymes may influence plant–pathogen interactions by modulating cell wall properties including thickness, rigidity, and structural integrity. Notably, we identified a legume-specific clade (Group III) in our phylogenetic analysis (Figure 1). Despite possessing conserved Glyco_hydro_3 and Glyco_hydro_3_C domains characteristic of xylan-hydrolyzing activity (Figure 4), the biological functions of these genes remain unexplored. Given the critical role of nodules in legume-specific symbiosis and their extreme sensitivity to salt stress, it is tempting to speculate that Group III *GmBXL* genes may play a role in nodule development or stress adaptation. For instance, these genes could be involved in modifying cell wall architecture during nodule formation or in mediating polysaccharide-derived signaling under stress conditions. Although further functional validation is needed, this hypothesis offers a compelling direction for future research aimed at understanding the mechanisms underlying nodule sensitivity to salt and the potential involvement of BXL-mediated cell wall remodeling in this process. As key rate-limiting enzymes in hemicellulose degradation, BXLs play crucial roles not only in cell wall remodeling but also in developmental processes. Previous studies have demonstrated BXL accumulation during fruit development, where they contribute significantly to fruit expansion [14,17]. Our findings reveal that in soybean, *GmBXL13* and *GmBXL26* exhibit strong high expression specificity in pods and seeds (Figure 6). Notably, *GmBXL26* shows nearly negligible expression in other tissues (Figure 6). These results suggest that *GmBXL13* and *GmBXL26* may serve specialized functions in seed development and potentially influence yield-related traits.

Salt stress represents a major environmental constraint that significantly impairs soybean yield and quality. This abiotic stress induces ROS accumulation, triggering oxidative stress that leads to cellular damage. To counteract these detrimental effects, soybean has evolved multiple defense mechanisms to mitigate ROS-induced oxidative stress. *E2*, the homolog of *A. thaliana GIGANTEA*, positively up-regulates the expressions of *ROS-scavenging* genes, thereby enhancing the salt tolerance of soybean [51]. *GmCONSTANS-like 1a* improves the salt tolerance by inhibiting the producing of ROS [46]. miR160a promotes salt tolerance by cleaving the transcript of the auxin response factor 16 (*GmARF16*) [52]. In turn, *GmARF16* activates *GmMYC2*, which encodes a bHLH transcription factor, thereby regulating the biosynthesis of proline and reducing the adaptability to salt stress [52]. Our study reveals that 26 *GmBXL* genes exhibit significant salt-responsive expression patterns in both leaves and roots (Figure 7). Notably, 24 genes showed marked up-regulation under salt stress, while *GmBXL8* and *GmBXL9* displayed leaf-specific down-regulation. These differential expression patterns strongly suggest that *GmBXL* genes participate in salt stress adaptation through cell wall component remodeling. Further investigation should focus on elucidating: (1) the precise regulatory mechanisms of *GmBXL* genes under salt stress, and (2) their specific roles in cell wall modification. These studies will provide crucial theoretical foundations for developing salt-tolerant soybean cultivars through molecular breeding approaches.

## 4. Materials and Methods

### 4.1. Plant Materials and Growth Conditions

The soybean reference cultivar Williams 82 (Wm82) was used for gene expression analyses. Plants were grown under long-day (16 h light/8 h dark) photoperiod in growth chambers (Conviron, Pembina, ND, USA/Winnipeg, MB, Canada) with 60% relative humidity at a constant temperature of 25 °C with a light intensity of 500 μmol m^−2^ s^−1^. Well-developed soybean seeds were germinated in vermiculite for 5 days, and then transferred to culture tanks containing Hoagland nutrient solution. When the plants reached V1 developmental stage, the treatment group was given 200 mM/L NaCl, and the control group was kept in NaCl-free nutrient solution. The roots and leaves were collected at the 4th hour after NaCl treatment. An air pump was used to maintain continuous aeration throughout the cultivation process, ensuring sufficient oxygen supply to the solution and promoting efficient root respiration.

### 4.2. Identification and Annotation of BXL Genes in Soybean

To obtain the BXL protein sequences in soybean, BLASTP was employed on the Phytozome 13 (https://phytozome-next.jgi.doe.gov/, accessed on 15 July 2025) [39], using the *A. thaliana* BXL protein sequences as a reference. Strict criteria were set for sequence alignment, requiring a minimum sequence homology of 60% and an *E*-value ≤ 1.0 × 10^−20^. Subsequently, the Hidden Markov Model (HMM) files of the BXL family (Glyco_hydro_3: PF00933, Glyco_hydro_3_C: PF01915, and Fn3-like: PF14310) were downloaded from the Pfam database (http://pfam.xfam.org/search, accessed on 15 July 2025). The HMM search plugin within TBtools software v2.337 was used to retrieve high-confidence genes in *G. max*, *M*. *truncatula* and *L*. *japonicus* [53]. All candidates for BXL proteins were confirmed using the CDD (https://www.ncbi.nlm.nih.gov/Structure/cdd/wrpsb.cgi, accessed on 15 July 2025) to filter out incomplete or divergent sequences. All gene names and sequences are listed in Tables S1 and S2.

### 4.3. Prediction of Physicochemical Properties of Soybean BXL Proteins

The number of amino acids, molecular weight, isoelectric point, instability index, aliphatic index and average hydrophobicity index of all identified soybean BXL proteins were analyzed by Prot Param in the online software ExPASy (https://web.expasy.org/protparam/, accessed on 15 July 2025) [54]. Plant-mPLoc (http://www.csbio.sjtu.edu.cn/bioinf/plant-multi/, accessed on 15 July 2025) was used to predict subcellular localization.

### 4.4. Phylogenetic Analysis of the BXL Proteins

The corresponding amino-acid sequences of the BXL proteins in *G. max*, *A. thaliana, M. truncatula* and *L. japinicus* were aligned using CLUSTALW to create multiple alignments on MEGA11 [40]. The phylogenetic tree was constructed using IQ-TREE version 2 [41] under the best-fit substitution model Q.plant + R5, as determined by ModelFinder [42]. Branch support was assessed using 1000 ultrafast bootstrap replicates. To further resolve the evolutionary relationships among soybean-specific *BXL* genes, a separate phylogenetic analysis was conducted using the protein sequences of all identified BXLs in *G. max*. Multiple sequence alignment was performed as described above, and the phylogenetic tree was reconstructed in IQ-TREE under the WAG + G4 model, which was determined to be optimal for this dataset. The resulting trees were visualized and annotated using the Interactive Tree of Life (https://itol.embl.de, accessed on 15 July 2025).

### 4.5. Chromosomal Localization and Collinearity Analysis

The chromosomal physical positions of *GmBXL* genes obtained from Phytozome database were visualized by importing the genome and gene position files into TBtools [53]. The chromosome distribution module in TBtools was also used to plot the gene positions for showing the respective chromosomes and relative positions and distances [55]. The soybean genome fasta file and corresponding GFF annotation file were downloaded from the Phytozome database. The One Step MCScanX plugin in TBtools was employed to analyze these files [56]. The results were visualized using the Circos visualization tool (https://circos.ca/) [57].

### 4.6. Gene Structure and Conserved Motifs and Domains of BXL Genes

According to the soybean gene feature format (GFF) file, the gene structure diagrams of the 29 members of *GmBXL* genes in soybean were constructed by TBtools software. The CDD platform was used to predict conserved domains of soybean BXL members. The MEME (https://meme-suite.org/meme/tools/meme, accessed on 15 July 2025) online tool was used to predict and analyze the conserved motifs [58]. The maximum number of motifs was set to 10, with other parameters set to their default values. Finally, the TBtools software was used to visualize the conserved motifs, and conserved domains [53].

### 4.7. Analysis of Cis-Acting Elements

The upstream sequences (2 Kb) of each *GmBXL* gene were obtained from the genome database. The PlantCARE program (http://bioinformatics.psb.ugent.be/webtools/plantcare/html/, accessed on 15 July 2025) was employed to identify putative *cis*-regulatory elements and visualized the types of elements and distribution within the upstream regions by TBtools [53,59]. The details of the *cis*-elements are listed in Table S4.

### 4.8. RNA-Seq Data Source and Expression Pattern Analysis of Soybean BXL Genes

According to Phytozome 13 (https://phytozome-next.jgi.doe.gov/, accessed on 15 July 2025), we selected FPKM (Fragments Per Kilobase of transcript per Million mapped reads) data for leaves, stem, shoot apex meristem (sam), flower, root, nodules, pod and seed. These RNA-seq data were originally generated by the Gary Stacey laboratory at the University of Missouri (http://staceylab.missouri.edu/, accessed on 15 July 2025) from the aforementioned tissues. A heatmap showed the expression patterns of soybean *BXL* genes using TBtools [53]. The FPKM values for *GmBXL* genes in different tissues are listed in Table S5.

### 4.9. RNA Extraction and Quantitative Real-Time PCR

Total RNA was extracted by an Ultrapure RNA Kit (CWBIO, Taizhou, China). cDNA was synthesized from 500 μg of RNA using a PrimeScript RT Reagent Kit with gDNA Eraser (Takara, Shiga, Japan). RT–qPCR was performed using a real-time PCR kit (cat. no. RR430; Takara) on a Roche Light Cycler 480 instrument II (Roche Molecular Biochemicals, Rotkreuz, Switzerland). Each 10 μL reaction contained 1 μL of 1:5 diluted cDNA with 0.2 μL of each primer (10 μM), 5 μL SYBR Green Master Mix, and water to a final volume of 10 μL. Each sample was assayed in triplicate with three biological replicates. The expression levels of target genes were normalized using *Tubulin* as the reference gene. The primers used in this experiment are listed in Table S6.

## 5. Conclusions

Salt stress represents a major abiotic constraint that severely limits soybean productivity. The identification of salt-responsive genes and elucidation of their molecular mechanisms are crucial for developing stress-resilient soybean cultivars. In this study, we systematically identified 29 *BXL* genes in soybean, whose genomic expansion was driven by both tandem and segmental duplications. Phylogenetic analysis categorized these genes into three distinct clades. Notably, promoter analysis revealed that most *GmBXL* genes contain abundant hormone-responsive *cis*-regulatory elements. Importantly, expression profiling demonstrated that 26 *GmBXLs* respond to salt stress, strongly implicating their functional involvement in salt stress adaptation. These findings provide valuable genetic resources and molecular insights for future investigations of *GmBXL* functions and significantly advance our understanding of soybean salt stress response mechanisms.

## Data Availability

All data generated or analyzed in this study are included in the main text and Supplementary Materials.

## Supplementary Materials

The following supporting information can be downloaded at: https://www.mdpi.com/article/10.3390/ijms26199534/s1.
