# Supplementary material for: Genome-Wide Identification of the BXL Gene Family in Soybean and Expression Analysis Under Salt Stress
Source: Int J Mol Sci. 2025 Sep 29;26(19):9534. doi: 10.3390/ijms26199534 (PMC12525369; doi:10.3390/ijms26199534)
Supplement: Supplementary file 1 [file ijms-26-09534-s001.zip › Supplementary Materials_S1.pptx]

## Slide 1
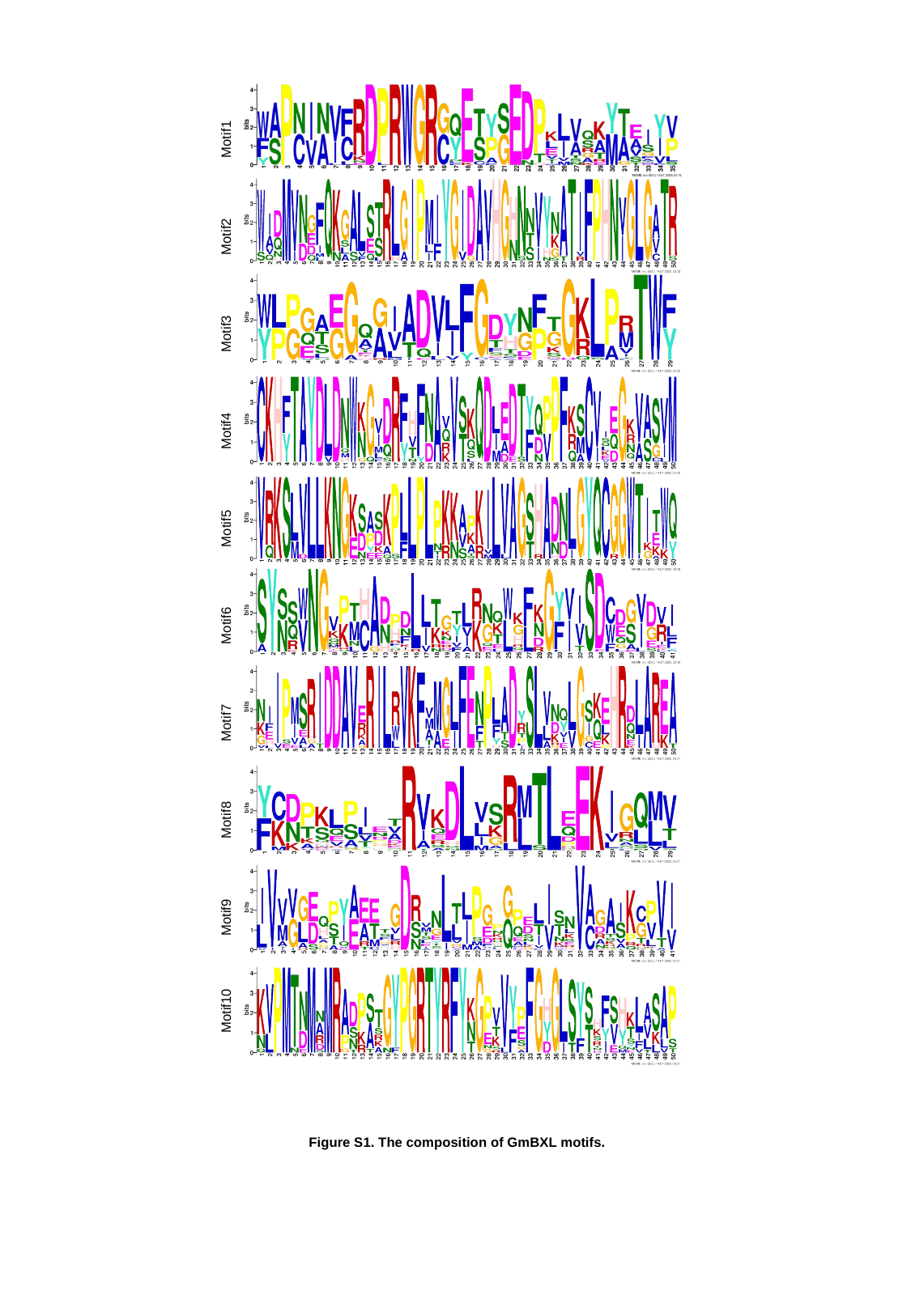

Motif1
Motif2
Motif3
Motif4
Motif5
Motif6
Motif7
Motif8
Motif9
Motif10
Figure S1. The composition of GmBXL motifs.
